# Comorbidities and mortality rate in COVID‐19 patients with hematological malignancies: A systematic review and meta‐analysis

**DOI:** 10.1002/jcla.24387

**Published:** 2022-04-06

**Authors:** Adel Naimi, Ilya Yashmi, Reza Jebeleh, Mohammad Imani Mofrad, Shakiba Azimian Abhar, Yasaman Jannesar, Mohsen Heidary, Reza Pakzad

**Affiliations:** ^1^ 56941 Cellular and Molecular Research Center Sabzevar University of Medical Sciences Sabzevar Iran; ^2^ 56941 Student Research Committee Sabzevar University of Medical Sciences Sabzevar Iran; ^3^ 56941 Department of Laboratory Sciences School of Paramedical Sciences Sabzevar University of Medical Sciences Sabzevar Iran; ^4^ Department of Epidemiology Faculty of Health Ilam University of Medical Sciences Ilam Iran

**Keywords:** COVID‐19, leukemia, lymphoma, myeloma, review

## Abstract

**Introduction:**

The global pandemic of coronavirus disease 2019 (COVID‐19) is caused by the severe acute respiratory syndrome coronavirus 2 (SARS‐CoV‐2). It seems that there is an association between blood cancer and an increased risk of severe COVID‐19. This study aimed to review the literature reporting the COVID‐19 outcomes in patients with hematological malignancies.

**Material and methods:**

In this systematic review and meta‐analysis, Pubmed, Embase, and Web of Science databases were searched using the following keywords: COVID‐19, SARS‐CoV‐2, blood cancer, myeloma, lymphoma, and leukemia. All the published articles in English from January 1, 2019, until March 10, 2021 were collected and evaluated.

**Results:**

In total, 53 studies with 2395 patients were included based on inclusion criteria. Most of these studies took place in Spain (14.81%), followed by the USA (11.11%), China (9.26%), and the UK (9.26%). More than half of COVID‐19 patients with hematological malignancy were male (56.73%). Oxygen therapy played an important role in COVID‐19 treatment. Moreover, anticoagulant therapies such as enoxaparin and heparin were two great assists for these patients. Fever (74.24%), cough (67.64%), and fatigue (53.19%) were the most reported clinical manifestations. In addition, hypertension and dyslipidemia were the most common comorbidities. The mortality rate due to COVID‐19 in patients with hematological malignancies was 21.34%.

**Conclusion:**

This study demonstrated that hematologic cancer patients were more susceptible to a severe COVID‐19 than patients without blood cancer. Thus, the management of COVID‐19 in these patients requires much more attention, and their screening should perform regularly.

## Graphical abstract

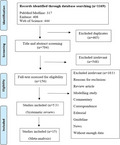

## 1 INTRODUCTION

The severe acute respiratory syndrome coronavirus 2 (SARS‐CoV‐2) caused the current global pandemic of coronavirus disease 2019 (COVID‐19). Although most patients with COVID‐19 have mild symptoms, some have more severe manifestations.^1^ Recent findings have suggested an association between cancer and an increased risk of developing severe symptoms of COVID‐19.^2^, ^3^, ^4^ Dai et al. reported that about 39% of the COVID‐19 patients with cancer had severe events such as intensive care unit admission, the need for mechanical ventilation, and even death. They showed that only 8% of the COVID‐19 patients without cancer had those severe symptoms.^5^ In addition, hematologic cancer patients with COVID‐19 had a high frequency of severe events like a higher mortality rate and a more severe COVID‐19 course.^6^ The immune system dysfunction is one of the main reasons that confirm patients with hematological malignancies are more vulnerable.^7^ Moreover, anti‐cancer therapies such as chemotherapy, radiotherapy, and immunosuppressive drugs worsen the condition of these patients.^8^ There are a limited number of studies on the prevalence of comorbidities and mortality rate in COVID‐19 patients with hematological malignancies. Therefore, in this systematic review and meta‐analysis, we will comprehensively review the available published literatures reporting the COVID‐19 outcomes and underlying diseases in patients with hematological malignancies from around the world.

## 2 MATERIALS AND METHODS

This study was performed following the “Preferred Reporting Items for Systematic Reviews and Meta‐Analyses” (PRISMA) statements.^9^

### 2.1 Search strategy

The Pubmed/Medline, Embase, and Web of Science databases, from January 1, 2019, until March 10, 2021, were searched to collect the potentially relevant articles reporting COVID‐19 disease in patients with hematological malignancies. The search was limited solely to publications in English.

The following keywords or Medical Subject Headings (MESH) terms were used in text, title, or abstract with the help of Boolean operators (“and,” “or”): “COVID‐19,” “severe acute respiratory syndrome coronavirus 2,” “SARS‐CoV‐2,” “nCoV disease,” “2019‐nCoV,” “coronavirus disease 2019,” “bone marrow cancer,” “blood cancer,” “myeloma,” “lymphoma,” “Waldenstrom macroglobulinemia,” “leukemia,” “hematological malignancy,” “myelodysplastic syndrome,” and “myeloproliferative disorder.”

### 2.2 Study selection

All the articles reporting COVID‐19 positive patients with at least one type of hematological malignancies were included. In other words, patients with blood cancer infected with the SARS‐CoV‐2 were enrolled in the study. The allogeneic stem cell transplantation patients were included as well. According to World Health Organization (WHO) guidelines, COVID‐19 cases are defined as patients whose reverse transcription‐polymerase chain reaction (RT‐PCR) is positive. Duplicate publications, narrative reviews, meta‐analyses, systematic reviews, editorials, correspondences, guidelines, articles published in languages other than English, and publications without enough data or available only in abstract form were also excluded. The included studies were screened in two stages for eligibility. First, title/abstract screening was done, and then, the full text of those that had the inclusion criteria was retrieved. It is worth noting that although we reviewed case‐report articles to evaluate some variables, only research articles and case series were included for meta‐analysis.

### 2.3 Data extraction

The extracted data included the first author’s name, country of the study, published time, type of study, number of patients, median age, gender, hematological malignancy type, blood cancer therapy, the median duration of blood cancer, COVID‐19 diagnosis method, COVID‐19 therapy, clinical manifestations, laboratory findings, comorbidities, and outcome. Two authors independently applied the inclusion criteria to the potentially relevant article, and discrepancies between the authors were resolved by consensus discussion.

### 2.4 Quality assessment

The quality assessment of the studies was carried out through the critical appraisal checklist provided by the Joanna Briggs Institute (JBI).^10^

### 2.5 Meta‐analysis

Data were analyzed using STATA software, version 17.0. The fixed‐effects model and random‐effects model were used to compute pooled estimates of the relative risk. The heterogeneity was quantified by the Cochran *Q* statistic and *I* ^2^ statistical methods. The *p*‐value <.05 was considered statistically significant.^11^

## 3 RESULTS

### 3.1 Characteristics of included studies

**FIGURE 1:**
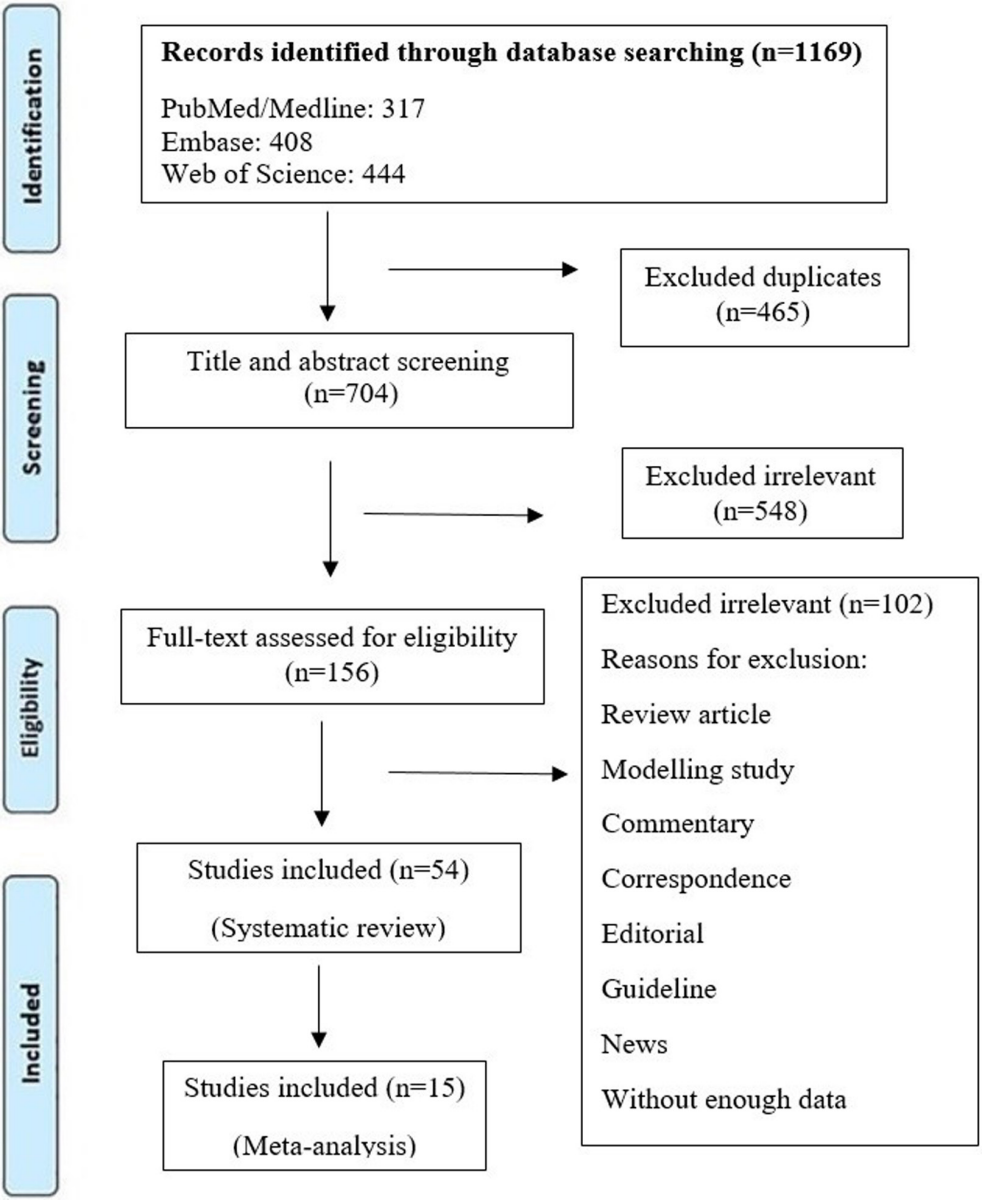
Flow diagram detailing review process and study selection

Initially, a total of 1169 articles were collected from databases. After removing the duplicates, 704 studies remained. In the screening phase, 548 of them were excluded through the title and abstract evaluation. Out of these studies, 53 met the inclusion pellucid criteria based on the full‐text screening. At the final stage, 15 eligible articles were included in the meta‐analysis (Figure 1). Characteristics of the selected articles are summarized in Table 1. Most of the studies took place in Spain (8/53, 14.81%), followed by the United States (6/53, 11.11%), China (5/53, 9.26%), and the United Kingdom (5/53, 9.26%).

**TABLE 1:** Characteristics of the included studies

First author	Country	Publish time	Type of study	No. of patients with blood cancer & COVID‐19	Median age	Male/female	Type of blood cancer	Treatment of blood cancer	Median duration of Blood cancer	SARS‐CoV‐2 diagnosis method	COVID‐19 treatment and ancillary medications	Clinical manifestations	Laboratory findings	Other comorbidities	Outcomes
Santana^33^	Brazil	Feb 2021	Case report	1	47	F	Grade 3A follicular lymphoma	rituximab, cyclophosphamide, vincristine, and PRED	NR	RT‐PCR, CT scans	mPDRL, oxygen support	Dyspnea, hypoxemia	Increased: D‐dimer, CRP	organizing pneumonia	Discharged
Ali^34^	Qatar	Oct 2020	Case report	1	49	M	CLL	None	NR	RT‐PCR, CT scans	OTV, AZ, HCQ, IV CRO, amoxicillin/clavulanate	Fever, mild dyspnea, body aches	Increased: WBC, ANC, lymphocyte, monocyte, ferritin, CRP Decreased: Albumin	None	Discharged
Nesr^35^	UK	Sep 2020	Case report	1	80	F	CLL	NR	NR	RT‐PCR, CT scans	IV DOX, oxygen support	Fever, cough, dyspnea	Increased: Lymphocyte, reticulocyte, LDH, bilirubin, CRP Decreased: Hb	congestive cardiac failure, atrial fibrillation, AIHA	Discharged
Molina‐Cerrillo^36^	Spain	Jan 2021	Case report	1	72	M	CLL	IBR	4y	RT‐PCR, CT scans	Oxygen support, HCQ, LPV/r	Cough, sore throat, fever	Increased: D‐dimer, CRP, LDH, lymphocyte, ferritin, IL‐6, IL‐8	Hypertension, dyslipidemia	Discharged
Largeaud^37^	France	Nov 2020	Case report	1	83	M	CLL	NR	NR	RT‐PCR, CT scans	Paracetamol, amoxicillin, clavulanic acid, corticosteroid therapy, anticoagulant therapy, oxygen support	Fever, cough, dyspnea, rectal bleeding	Increased: CRP Decrease: Hb, WBC, ANC, lymphocyte, PLT	radiotherapy treated pulmonary neoplasia	Discharged
Bolaman^38^	Turkey	Feb 2021	Case report	1	NR	F	DLBCL	Chemotherapy	NR	RT‐PCR, CT scans	HCQ, AZ, favipiravir, oxygen support	Cough, dyspnea, orthopnea	Increased: Fibrinogen, D‐dimer, LDH Decreased: Hb, WBC, lymphocyte	NR	Death
Pasin^39^	Africa	Jul 2020	Case report	1	20	M	Refractory NK/T‐cell lymphoma	rituximab, pembrolizumab, l‐asparaginase	NR	RT‐PCR, CT scans	RBC transfusions, mPDRL, Oxygen support, IV levofloxacin, supportive therapy, steroid therapy	Fatigue, fever, cough, dyspnea	Increased: WBC, CRP, LDH, indirect bilirubin Decreased: Hb, PLT	EBV, AIHA	Discharged
Ibrahim^40^	Saudi Arabia	Sep 2020	Case report	1	57	M	CML	Imatinib	10y	RT‐PCR, CT scans	Paracetamol and antitussive‐ HCQ, AZ, CRO, enoxaparin, oxygen support, mPDRL, LPV/r, ribavirin, IFN	Cough, fever, nausea	Increased: WBC, ANC, lymphocyte, D‐dimer, ferritin, LDH	Diabetes mellitus	Discharged
Chaidos^41^	UK	May 2020	Case series	2	62.5	M 2	MM 2	lenalidomide 1, bortezomib 1, panobinostat 1	NR	RT‐PCR, CT scans	Tocilizumab 2, oxygen support 1	Fever 2, cough 1, hypoxia 2	Increased: CRP 2, ferritin 2, D‐dimer 2	NR	Discharged 2
O’Kelly^42^	Ireland	May 2020	Case report	1	22	F	HL	ABVD, BEACOPP, ICE, brentuximab vedotin, IFRT, pembrolizumab	4y	RT‐PCR, CT scans	TZP, DOX, LPV/r, antibiotics, HCQ, AZ, oxygen support, corticosteroids	Cough, fever, sore throat, chills, rigors	Increased: CRP, LDH Decreased: Lymphocyte, PLT	NR	Discharge
Day^43^	UK	May 2020	Case series	3	35.6	M 3	AML 2, ALL 1	daunorubicin 2, cytarabine 2, gemtuzumab ozogamicin 2, blinatumomab 1	less than 1 y	RT‐PCR, CT scans	antibiotics 3, anakinra 3, IVIg 2, oxygen support 3	Cough 2, rhinorrhea 1, sore throat 1, diarrhea 2, fever 3, rash 2, dyspnea 1	Increased: Ferritin 3, triglycerides 3, CRP 1 Decreased: PLT 3, RBC 2, WBC 2, lymphocyte 1	Previous seizures	Discharged 3
Bellmann‐Weiler^44^	Austria	Jun 2020	Case series	3	65	M 3	AML 1, follicular lymphoma 1, hairy cell leukemia 1	Bendamustine 1, rituximab 1	8y	RT‐PCR, CT scans	Oxygen support 3, physiotherapy 1, HCQ 1, AZ 1, favipiravir 2, antibiotics 1	Fever 2, dyspnea 2, cough 3, asthenia 2, anorexia 1, diarrhea 1	Increased: CRP, IL‐6 Decreased: WBC 3, lymphocyte 3, Hb, PLT	diabetes 1, hypertension 2, obesity 3, coronary heart disease 1	Discharged 3
Susek^45^	Sweden	Aug2020	Original article	9	70.4	M 6 F 3	MM 8, smoldering MM 1	daratumumab 6, DEX 8, venetoclax 1, carfilzomib 1, bortezomib 1, lenalidomide 3	NR	RT‐PCR	Oxygen support 4	Fever 9, cough 8, dyspnea 3, diarrhea3, arthralgia3, ageusia 3	Increased: CRP 5 Decreased: Hb 9, WBC 3, ANC 2, lymphocyte	Diabetes 4, hypertension 3, obesity 2	Discharged 5 Died 4
Ye^46^	China	Jul 2020	Case report	1	72	F	CLL	NR	NR	RT‐PCR, CT scans	LPV/r, IFN, IVIg, ARB	Fever, cough	Increased: Lymphocyte, D‐dimer	NR	Discharged
Phillips^47^	US	Sep 2020	Case report	1	18	M	ALL	vincristine, daunorubicin, mPDRL	NR	RT‐PCR	Oxygen support, corticosteroids, vasopressor	Fever, cough	Increased: WBC, LDH Decreased: Hb, PLT, hyperuricemia	AIHA	Discharge
Zamani^48^	Iran	Jan 2021	Case report	1	35	F	AML	Chemotherapy	less than 1 y	RT‐PCR, CT scans	NR	Dyspnea, malaise, cough	NR	AMN	Death
Krengli^24^	Italy	Dec 2020	Case report	1	62	F	MM	Bortezomib‐thalidomide‐DEX, cyclophosphamide, melphalan, radiotherapy, carfilzomib +DEX	2y	RT‐PCR, CT scans	HCQ, darunavir‐cobicistat, oxygen support	Cough, fever, dysphagia	Increased: CRP Decreased: Hb	hypercholesterolemia, osteoporosis	Death
Kohla^49^	Qatar	Dec 2020	Case report	1	58	M	Hairy Cell Leukemia	NR	0y	RT‐PCR, CT scans	HCQ, AZ, tocilizumab, mPDRL, IVIg, vasopressors, antibiotics, oxygen support	Fever, fatigue, cough, dyspnea	Increase: Creatinine, ALT, AST, LDH, IL‐6, D‐dimer, ferritin Decrease: WBC, ANC, Hb, PLT	None	NR
Engelhardt,^50^	Germany	2020 Jul	Cohort	21	59	M 17 F 4	MM	daratumumab‐combination 5, elotuzumab‐combination 1, VCd/KRd 2/1, lenalidomide 3, none 9	NR	RT‐PCR.	Antibiotics 17, AZ 4, HCQ 7, RDV 1, Tocilizumab 1, Anakinra 1, oxygen support 3	Cough 17, fever 16, myalgia 4, GI symptoms 2	NR	None 4, cardiac/ hypertension 11, renal impairment 3, obesity 1, PNP 4, diabetes 4, hypothyreosis 4	Discharged 21
Rusconi^51^	Italy	July 2020	Case report	1	62	M	classical HL	ABVD	2 y	RT‐PCR, CT scans	Levofloxacine, oxygen support, HCQ, LPV/r, enoxaparin, tocilizumab, CRO	Fever	Increased: Creatinine, fibrinogen, D‐dimer, CRP, LDH, ferritin Decreased: Lymphocyte	Hypertension, melanoma, papillary renal cell cancer	Discharged
Denis^52^	France	Jul 2020	Case report	1	72	F	Mantle cell lymphoma	R‐CHOP	NR	RT‐PCR, CT scans	Kaletra, CRO	Confusion	NR	NR	NR
Moore^53^	US	Oct 2020	Case report	1	63	F	non‐HL	obinutuzumab	NR	RT‐PCR	Plasma	Fever, myalgia, cough	Increased: CRP, LDH Decreased: WBC, lymphocyte	NR	Discharged
Vardanyan^54^	UK	Jul 2020	Case report	1	61	F	CLL	NR	NR	RT‐PCR, CT scans	Oxygen support, amoxicillin‐clavulanic acid, TZP, clarithromycin, tocilizumab	Fever, dyspnea, cough, fatigue	Increased: D‐dimer, ferritin Decreased: Hb, WBC, lymphocyte	NR	NR
Abdalhadi^55^	Qatar	May 2020	Case report	1	65	M	CML	Dasatinib	4 y	RT‐PCR, CT scans	HCQ, AZ, OTV, TZP, oxygen support, LPV/r, tocilizumab, mPDRL	Fever, cough, chest pain	Increased: D‐dimer, CRP, LDH Decreased: ANC, Hb, PLT	NR	Discharged
Giammarco^56^	Italy	Dec 2020	Case report	1	50	M	AML	all‐trans PRED	NR	RT‐PCR, CT scans	NR	Fever, ostealgia	Increased: LDH, creatine kinase, D‐dimer Decreased: ANC, PLT	None	Death
Li^57^	China	Dec 2020	Case report	1	61	M	MM	bortezomib, DEX	0 y	RT‐PCR, CT scans	Ceftazidime, oxygen support, IVIg, meropenem, teicoplanin, Ganciclovir, IFN, ARB, OTV, moxifloxacin	Fever, cough, chest pain, dyspnea	Increased: CRP, D‐dimer Decreased: Hb, lymphocyte	NR	Discharged
Marcia^58^	Italy	Jul 2020	Case report	1	3	M	ALL	PRED, vincristine‐daunorubicin	NR	RT‐PCR, CT scans	Antibiotics, LPV/r, HCQ	Fever, epistaxis, weight loss, bruises, hepatosplenomegaly	Increased: WBC Decreased: Hb, PLT	NR	Discharged
Kamit^59^	Turkey	Nov 2020	Case report	1	9	F	ALL	intrathecal‐IV methotrexate, vincristine, cyclophosphamide, cytosine arabinoside, L‐asparaginase, DEX	0.5 y	RT‐PCR, CT scans	vancomycin, meropenem, trimethoprim–sulfamethoxazole, ganciclovir, oxygen support, IVIg, Favipiravir, hydrocortisone, tocilizumab, plasma	Fever, cough	Decrease: WBC, lymphocyte, ANC	Angelman syndrome	Death
Otsuka^60^	Japan	Nov 2020	Case report	1	56	M	Mantle cell lymphoma	rituximab/cyclophosphamide/vincristine sulfate/doxorubicin, hydrochloride/DEX/methotrexate/cytarabine, bendamustine/rituximab	2 y	RT‐PCR, CT scans	Favipiravir, antibiotic, cefepime, oxygen support, HCQ, VAN, TZP, IVIg, ciclesonide, meropenem, teicoplanin	Fever	Increased: AST, ALT, Decreased: WBC, lymphocyte, Hb, PLT	NR	Death
Bellesso^61^	Brazil	Mar 2021	Case report	1	76	F	MM	Bortezomib, DEX, radiotherapy, daratumumab	1.5 y	RT‐PCR	CRO, VAN, oxygen support, vasoactive drug, meropenem	Confusion, hip pain, respiratory distress	NR	ESRD, hypertension, glucose intolerance	Death
Glenthøj^62^	Denmark	Sep 2020	Cohort	66	66.7	M 40 F 26	MM 11, CLL 31, AML 8	rituximab 14, daratumumab 4, purine analogues 7, ibrutinib 3, non‐cancer immunosuppressive treatment 5	NR	RT‐PCR, CT scans	Oxygen support 42	Fever 53, Cough 50, Dyspnea 22, Headache 11, Myalgia 6, Diarrhea 3	Decreased: lymphocyte 27, ANC 4	Obesity 8, smokers 3, heart disease 3, lung disease 9, diabetes 9, renal disease 7, liver disease 1	Discharged 50 Death 16
Wang^63^	US	July2020	Cohort	58	67	M 30 F 28	MM 54, smoldering MM 4	daratumumab 28, immunomodulatory drugs 32, proteasome inhibitor 22, venetoclax 5, corticosteroids 30	2 y (29.8 months)	RT‐PCR	oxygen support 10, RDV 1, HCQ 17, AZ 17, antibiotics 19, corticosteroid 10, plasma 1, selinexor 5, anti‐IL‐6 4, anti‐IL‐1 2, anti TNF 1	Fever 40, Cough 37, dyspnea 26	Decreased: WBC 20, ANC 15, lymphocyte 7	Hypertension 37, Hyperlipidemia 36, Obesity 21, Diabetes 16, chronic kidney disease 14, lung disease 12, current or former smoker 21, CAD and/or CVD 13, heart failure 7	44 Discharged Death 14
Sánchez‐Jara^64^	Mexico	Mar2021	Original article	15	7.5	M 8 F 7	ALL 12, AML 3	Chemotherapy	NR	RT‐PCR, CT scans	Oxygen therapy 13	Fever 13, rhinorrhea 2, cough 9, headache 4, respiratory distress 8, seizures 1, irritability 4, sore throat 2, diarrhea 2, drowsiness 2	Increased: CRP 13 Decreased: ANC 13, RBC 13, WBC 13, lymphocyte 14, PLT 13	NR	Discharged 8 Death 7
Garcia‐Suarez^65^	Spain	Oct 2020	Observational study	697	72	NR	non‐HL 187, MM 136, CLL 109, HL 32, ALL 13, myelodysplastic syndrome 78, AML 61, CML 16, Ph‐negative myeloproliferative neoplasms 63	Chemotherapy 169, molecular targeted therapies 81, immunomodulatory drugs 45, monoclonal antibodies 44, Hypomethylating agents 33, none 286	NR	RT‐PCR	HCQ 558, AZ 276, antiretrovirals 337, IFN 50, corticosteroid 318, tocilizumab 132	NR	NR	Hypertension 277, cardiac disease 138, diabetes 121, renal disease 77, pulmonary disease 90	Discharged 467 Death 230
Martinez‐Lopez^66^	Spain	Oct 2020	Case series	167	71	M 95 F 72	MM 167	Proteasome inhibitor 138, immunomodulatory drug 119, monoclonal antibody 38	>18 m 112, <18 m 55	RT‐PCR	HCQ 148, AZ 91, antiretrovirals 103, steroids 83, Anti‐interleukin‐6 receptor antibody therapy 22, heparin 109, oxygen support 128	NR	NR	None 41, cardiac disease 35, pulmonary disease 23, diabetes 28, renal disease 32, hypertension 67	Discharged 111 Death 56
Regalado‐Artamendi^67^	Spain	Feb 2021	Original article	177	70	M 99 F 78	HL 19, follicular lymphoma 62, DLBCL 39, other aggressive lymphomas 27, other indolent lymphomas 30	CD20‐chemotherapy 58, CD20‐bendamustine 20, Chemotherapy 33, Molecular targets 3, Immunotherapy 38	NR	RT‐PCR	LPV/r 89, HCQ 156, IFN 13, AZ 79, RDV 9, plasma 7, tocilizumab 51, anakinra 11, mPDRL 65, DEX 20, oxygen support 125	Fever 134, cough 115, dyspnea 87, myalgia 45, diarrhea 36, chest pain 25, rhinorrhea 15, anosmia 14, sore throat 7	NR	Heart disease 34 Hypertension 73 Diabetes 33, Obesity 14, Dyslipidemia 27, Chronic pulmonary disease 23, Asthma 9, Chronic kidney disease 11, Chronic liver disease 4	Discharged 116 Death 61
Yigenoglu^17^	Turkey	Aug2020	Cohort	740	56	M 397 F 343	HL 27, CLL 54, MM 77, ALL 18, myeloproliferative neoplasm 116, CML 30, non‐HL 223, Myelodysplastic syndrome 146, AML 40, hairy cell leukemia 9	NR	NR	RT‐PCR	Favipiravir 189, OTV 309, LPV/r 35, HCQ 508	NR	NR	Hypertension 379, diabetes 198, cardiovascular disease 156, respiratory disease 175	Discharged 701 Death 39
Piñana^68^	Spain	Aug 2020	Observational study	367	64	M 225 F 142	Non‐HL 91, AML 67, ALL 25, Myelodysplastic syndrome 22, chronic myeloproliferative disease 29, CLL 4	NR	NR	RT‐PCR	AZ 156, HCQ 147, LPV/r 163, RDV 8, corticosteroid 10, tocilizumab 50, anakinra 18, baricitinib 7	None 30, fever 259, rhinorrhea 54, pharyngitis 27, fatigue 196, myalgia 73, cough 244, diarrhea 81, vomiting 37	Increased: CRP 200, D‐dimer 172, ferritin 119 Decreased: ANC 48, lymphocyte 140	Smoking 33, hypertension 142, cardiomyopathy 65, dyslipidemia 94, diabetes 86	Discharged 262 Death 105
de la Cruz‐Benito^69^	Spain	August 2020	Cohort	1	52	F	DLBCL	R‐CHOP	NR	RT‐PCR	NR	NR	Decreased: Lymphocyte	Dyslipidemia	NR
Başcı^70^	Turkey	July2020	Original article	16	51	M 6 F 10	CML 16	Imatinib 9, Nilotinib 3, Dasatinib 4	NR	RT‐PCR	Favipiravir 4, OTV 9, LPV/r 1, HCQ 13	NR	NR	None 5, COPD 4, diabetes 3, hypertension 7, CAD 5, chronic renal disease 2, CVD 1	NR
Naseri^71^	Iran	Oct 2020	Case report	1	42	F	AML	Idarubicin, cytarabine	new	RT‐PCR, CT scans	Oxygen support, linezolid, meropenem, LPV/r, IFN	Fever, dyspnea, myalgia	Increased: CRP, ferritin, LDH, D‐dimer Decreased: WBC, Hb, PLT, ANC, lymphocyte	Diabetes	Death
Song^72^	China	Dec 2019	Case report	1	78	F	CLL	None	5 y	RT‐PCR, CT scans	OTV, cefoperazone, sulbactam, linezolid, mPDRL, oxygen support	Fatigue, malaise, hyporexia	Increased: WBC, lymphocyte, CRP Decreased: Hb	Hypertension, cardiovascular disease, COPD	Death
Li^73^	China	May 2020	Case report	1	26	M	B‐cell lymphoma	DA‐EPOCH‐R	NR	RT‐PCR, CT scans	Meropenem, linezolid, AZ, ganciclovir, OTV, ARB	Fever	Decreased: ANC, lymphocyte	None	Discharged
Baldacini^74^	France	May 2020	Case report	1	62	F	AML	NR	less than 1 y	RT‐PCR, CT scans	NR	Asthenia, dyspnea, epistaxis	Increased: WBC, CRP, D‐dimer Decreased: Hb, PLT, ANC	NR	Death
Farmer^75^	UK	Jun 2020	Case report	1	36	M	AML	Arsenic trioxide	less than 1 y	RT‐PCR	NR	Fever, cough, sweats	Increased: D‐dimer, ferritin, creatinine, LDH, CRP Decreased: WBC, Hb, ANC, lymphocyte, PLT	NR	NR
Puyo^76^	Spain	Jan 2020	Case report	1	20 months	M	ALL	Chemotherapy	2 months	RT‐PCR	TZP, amikacin, oxygen support, HCQ, AZ, VAN, tocilizumab	Fever	Decreased: ANC	NR	Discharged
Malek^77^	US	Jul 2020	Case report	1	41	F	CLL	NR	NR	RT‐PCR, CT scans	Cefepime, linezolid, DOX, mPDRL, oxygen support	Fever, nausea, vomiting, diarrhea, cough, dyspnea, myalgia	Increased: WBC, Lymphocyte, ALT, AST, CRP, D‐dimer, LDH, ferritin	Obesity	Discharged
Schied^78^	US	Sep 2020	Case report	1	6	F	B lymphoblastic lymphoma	Chemotherapy	NR	RT‐PCR	Supportive care	None	Increased: Ferritin	NR	Discharged
Pandrowala^79^	US	Mar 2021	Case report	1	5	F	AML	Daunorubicin, cytarabine, fludarabine, idarubicin, ventoclax, 5‐azacytidine	NR	RT‐PCR	Oxygen support, mPDRL, meropenem, amikacin	Fever	Increased: WBC, CRP Decreased: Hb, ANC, PLT	NR	Discharged
Rathore^80^	India	Jun 2020	Case report	1	10	M	ALL	Chemotherapy	3 months	RT‐PCR	DEX	Cough	NR	NR	Discharged
Zhang^81^	China	Apr 2020	Case report	1	60	M	MM	Bortezomib, thalidomide, DEX	5 y	RT‐PCR, CT scans	Moxifloxacin, ARB, oxygen support	Chest tightness, dyspnea	Increased: CRP Decreased: Lymphocyte	NR	Discharged
Ghandili^32^	Germany	Dec 2020	Case series	12	60	M 9 F 3	AML 8, ALL 3, lymphoblastic lymphoma 1	NR	NR	RT‐PCR	Oxygen support 5, LPV/r 1, pentaglobin 2, plasma 1, tocilizumab 1, None 5	None 4	Decreased: ANC 12, lymphocyte 12	Hypothyroidism 1, Asthma 1, allergic rhinitis 1, smoker 1	Discharged 10 Death 2
Kos^82^	Germany	Sep 2020	Case report	1	72	M	Marginal zone lymphoma	Bendamustine, rituximab	NR	RT‐PCR	Ampicillin, sulbactam, meropenem, clarithromycin, IVIg	Fever, cough	Increased: CRP, LDH Decreased: Hb	None	Discharged
Nunez Torron^83^	Spain	Jun 2020	Cohort	4	54.5	M 3 F 1	AML 4	Chemotherapy	new	RT‐PCR, CT scans	HCQ 4, LPV/r 3, AZ 1, corticosteroids 3, tocilizumab 2, oxygen support 4	Fever 4, cough 1, asthenia 1	Increased: WBC 1 Decreased: Hb 1, PLT 1	None 4	Discharged 1 Death 3

### 3.2 Demographic, clinical and laboratory findings

The demographic information, clinical features, and laboratory findings in COVID‐19 patients with hematological malignancies are shown in Tables 2 and 3. The results of laboratory data showed that ALT, AST, CRP, and LDH tests have increased in COVID‐19 patients with hematological malignancies. However, hemoglobin level, platelet count, lymphocyte count, and RBC decreased in these patients. The majority of patients were male (56.73%). Fever (74.24%), cough (67.64%), and fatigue (53.19%) were the most common clinical manifestations among the included patients.

**TABLE 2:** Summary of the findings in COVID‐19 patients with hematological malignancies

	*n*/*n* (%)	No. of studies that mentioned
Gender		
Male	962/1698 (56.65%)	53
Female	736/1698 (43.35%)	
Treatment of blood cancer		
Proteasome inhibitor	160/521 (30.71%)	5
Chemotherapy	209/891 (23.46%)	11
Immunotherapy	239/1175 (20.34%)	7
Monoclonal antibodies	140/1051 (13.32%)	6
Daratumumab	44/442 (9.95%)	7
Molecular targeted therapy	84/884 (9.50%)	2
Corticosteroids	47/371 (12.66%)	13
Rituximab	19/370 (5.14%)	10
Bendamustine	23/478 (4.81%)	7
Hypomethylating agents	33/707 (4.67%)	3
Imatinib	10/313 (3.19%)	5
Lenalidomide	7/319 (2.19%)	5
Purine analogues	7/362 (1.93%)	4
Bortezomib	6/311 (1.92%)	9
Ventoclax	7/364 (1.91%)	6
Vincristine	5/301 (1.66%)	6
Cytarabine	5/302 (1.65%)	7
Daunorubicin	5/302 (1.65%)	7
Dasatinib	5/313 (1.60%)	2
Cyclophosphamide	4/301 (1.33%)	8
Nilutinib	3/312 (0.96%)	4
Ibrutinib	3/362 (0.82%)	4
ABVD	2/298 (0.67%)	5
Idarubicin	2/298 (0.34%)	5
L‐asparginase	2/298 (0.67%)	5
Methotrexate	2/298 (0.67%)	5
Pembrolizumab	2/298 (0.67%)	5
Radiotherapy	2/298 (0.67%)	5
R‐CHOP	2/298 (0.67%)	5
Thalidomide	2/298 (0.67%)	5
Gemtuzumab ozogamicin	2/299 (0.66%)	4
Carfilzomib	2/306 (0.65%)	5
VCd	2/308 (0.64%)	4
5‐Azacytidine	1/297 (0.34%)	4
Arsenic trioxide	1/297 (0.34%)	4
BEACOPP	1/297 (0.34%)	4
Brentuximab	1/297 (0.34%)	4
Cytosine arabinose	1/297 (0.34%)	4
DA‐EPOCH	1/297 (0.34%)	4
Doxorubicin	1/297 (0.34%)	4
Hydrochloride	1/297 (0.34%)	4
Fludarabine	1/297 (0.34%)	4
ICE	1/297 (0.34%)	4
IFRT	1/297 (0.34%)	4
Melphalan	1/297 (0.34%)	4
mPDRL	1/297 (0.34%)	4
Obinutuzumab	1/297 (0.34%)	4
Panobinostat	1/297 (0.34%)	4
Vedotin	1/297 (0.34%)	4
Blinatumomab	1/299 (0.33%)	4
Elotuzumab	1/308 (0.32%)	4
KRd	1/308 (0.32%)	4
COVID‐19 treatment & ancillary medications		
Hydroxychloroquine (HCQ)	1571/2267 (69.30%)	23
Oxygen support	365/559 (65.30%)	36
Anticoagulant therapy	112/175 (64%)	5
Meropenem	8/13 (61.54%)	9
IV immunoglobulin (IVIg)	8/14 (57.14%)	8
Antiretrovirals	440/869 (50.63%)	2
Ceftriaxone (CRO)	5/10 (50%)	6
Piperacillin/tazobactam (TZP)	5/10 (50%)	6
Antibiotics	44/94 (46.81%)	8
Arbidol (ARB)	4/9 (44.44%)	5
Linezolid	4/9 (44.44%)	5
Vancomycin (VAN)	4/9 (44.44%)	5
Corticosteroid therapy	429/1009 (42.52%)	10
Oseltamivir (OTV)	323/766 (42.17%)	8
Azithromycin (AZ)	633/1502 (42.14%)	16
Methylprednisolone (mPDRL)	73/190 (38.42%)	11
Amoxicillin/clavulanate	3/8 (37.50%)	4
Doxycycline (DOX)	3/8 (37.50%)	4
Ganciclovir	3/8 (37.50%)	4
Vasoactive drugs (including vasopressor)	3/8 (37.50%)	4
Supportive therapy	3/10 (30%)	4
Amikacin	2/7 (28.57%)	3
Cefepime	2/7 (28.57%)	3
Clarithromycin	2/7 (28.57%)	3
Levofloxacin	2/7 (28.57%)	3
Moxifloxacin	2/7 (28.57%)	3
Paracetamol	2/7 (28.57%)	3
Sulbactam	2/7 (28.57%)	3
Teicoplanin	2/7 (28.57%)	3
Favipiravir	198/830 (23.85%)	7
Lopinavir/ritonavir (LPV/r)	300/1324 (22.66%)	14
Tocilizumab	245/1286 (19.05%)	13
Ampicillin	1/6 (16.67%)	2
Cefoperazone	1/6 (16.67%)	2
Ceftazidime	1/6 (16.67%)	2
Ciclesonide	1/6 (16.67%)	2
Darunavir‐cobicistat	1/6 (16.67%)	2
Kaletra	1/6 (16.67%)	2
Pentaglobin	2/12 (16.67%)	1
RBC transfusion	1/6 (16.67%)	2
Ribavirin	1/6 (16.67%)	2
Trimethoprim–sulfamethoxazole	1/6 (16.67%)	2
Anti‐IL‐6	26/240(10.83%)	3
Selinexor	5/63 (7.93%)	2
Interferon (IFN)	67/883 (7.59%)	6
Anakinra	33/573 (5.76%)	5
Plasma	11/249 (4.42%)	5
Anti‐IL‐2	2/63 (3.17%)	2
Remdesivir (RDV)	19/628 (3.03%)	4
Baricitinib	7/372 (1.88%)	2
Anti TNF	1/63 (1.59%)	2
Clinical manifestations		
Fever	562/757 (74.24%)	38
Cough	508/751 (67.64%)	30
Fatigue	200/376 (53.19%)	7
Dyspnea	155/366 (42.34%)	23
Myalgia	131/639 (20.50%)	7
Respiratory distress	9/51 (17.65%)	5
Diarrhea	48/309 (15.53%)	10
Chest pain or tightness	28/215 (13.02%)	7
Headache	15/116 (12.93%)	5
Rhinorrhea	72/567 (12.70%)	6
Vomiting	38/373 (10.19%)	4
Asthenia	4/43 (9.30%)	6
Irritability	4/50 (8%)	4
Pharyngitis	27/372 (7.26%)	3
Ageusia	3/44 (6.82%)	4
Arthralgia	3/44 (6.82%)	4
Anosmia	14/212 (6.60%)	4
Confusion	2/37 (5.41%)	5
Epistaxis	2/37 (5.41%)	5
Hypoxia	2/37 (5.41%)	4
Malaise	2/37 (5.41%)	5
Nausea	2/37 (5.41%)	5
Rash	2/37 (5.41%)	4
Sore throat	12/232 (5.17%)	8
Anorexia or Hyporexia	2/39 (5.13%)	5
Drowsiness	2/50 (4%)	4
Gastrointestinal symptoms	2/56 (3.57%)	4
Body aches	1/36 (2.78%)	4
Bruises	1/36 (2.78%)	4
Chills	1/36 (2.78%)	4
Dysphagia	1/36 (2.78%)	4
Hypoxemia	1/36 (2.78%)	4
Orthopnea	1/36 (2.76%)	4
Ostealgia	1/36 (2.78%)	4
Rectal bleeding	1/36 (2.78%)	4
Rigors	1/36 (2.78%)	4
Sweats	1/36 (2.78%)	4
Weight loss	1/36 (2.78%)	4
Seizures	1/50 (2%)	4
Laboratory findings		
Increased		
ALT	3/3 (100%)	3
ANC	2/2 (100%)	2
AST	3/3 (100%)	3
bilirubin	2/2 (100%)	1
Creatine kinase	1/1 (100%)	1
Creatinine	2/2 (100%)	2
Fibrinogen	2/2 (100%)	2
IL‐6	3/3 (100%)	2
IL‐8	1/1 (100%)	1
LDH	16/16 (100%)	16
Lymphocyte	7/7 (100%)	7
Monocyte	1/1 (100%)	1
Reticulocyte	1/1 (100%)	1
Triglycerides	3/3 (100%)	1
WBC	10/13 (76.92%)	10
CRP	242/417 (58.03%)	26
D‐dimer	189/384 (49.22%)	17
Ferritin	134/382 (35.08%)	13
Decreased		
Albumin	1/1 (100%)	1
Uric acid	1/1 (100%)	1
Hb	29/32 (90.62%)	21
PLT	31/36 (86.11%)	17
RBC	15/18 (83.33%)	2
WBC	50/97 (51.55%)	14
Lymphocyte	218/538 (40.52%)	20
ANC	105/538 (19.52%)	17

Abbreviations: ABVD, adriamycin, bleomycin, vinblastine, dacarbazine; AIHA, autoimmune hemolytic anemia; ALL, acute lymphoblastic leukemia; ALT, alanine aminotransferase; AML, acute myeloid leukemia; AMN, acute macular neuroretinopathy; ANC, absolute neutrophil count; ARB, arbidol; AST, aspartate aminotransferase; AZ, azithromycin; BEACOPP, bleomycin, etoposide, adriamycin, cyclophosphamide, vincristine, procarbazine, prednisolone; CHOP, cyclophosphamide, doxorubicin hydrochloride (hydroxydaunorubicin), vincristine sulfate (oncovin), and prednisone; CLL, Chronic lymphocytic leukemia; CML, chronic myelogenous leukemia; CRO, ceftriaxone; CRP, C‐reactive protein; CXR, chest X‐ray; DLBCL, diffuse large B‐cell lymphoma; DOX, doxycycline; EBV, Epstein‐Barr virus; HB, hemoglobin; HCQ, hydroxychloroquine; HGBL, high grade B‐cell lymphoma; HL, Hodgkin lymphoma; IBR, ibrutinib; ICE, ifosfamide, carboplatin, etoposide; IFN, interferon; IFRT, involved field radiotherapy; IV, intravenous; Ig, immunoglobulin; LDH, lactate dehydrogenase; LPV, lopinavir; LPV/r, lopinavir/ritonavir; MM, multiple myeloma; mPDRL, methylprednisolone; NR, not reported; OTV, oseltamivir; PLT, platelet; PRED, prednisone; RTV, ritonavir; RDV, remdesivir; TZP, piperacillin/tazobactam; VAN, vancomycin; VCd/KRd:bortezomib‐cyclophos‐phamid‐dexamethasone/carfilzomib‐lenalidomide‐dexamethasone.

**TABLE 3:** The main findings in COVID‐19 patients with hematological malignancies categorized based on the type of malignancy

	Total (%)	ALL	AML	CLL	CML	HL	NHL	MM	MDS	MPN
Patients	2395	77 (4.19)	200 (9.16)	214 (13.60)	64 (4.40)	80 (4.95)	614 (30.01)	486 (27.55)	246 (13.63)	208 (11.53)
Treatment of blood cancer										
Chemotherapy	209 (23.46)	20 (76.92)	28 (41.27)	4 (2.15)	2 (12.5)	21 (41.17)	83 (34.15)	33 (24.26)	2 (2.56)	31 (49.21)
Immunotherapy	239 (20.34)	–	–	–	–	7 (36.84)	20 (13.69)	196 (54.29)	–	–
Monoclonal antibodies	140 (13.32)	–	–	1 (0.54)	–	–	25 (13.37)	52 (17.17)	–	–
Molecular targeted therapy	84 (9.50)	–	3 (4.91)	28 (15.05)	14 (87.5)	–	8 (3.32)	12 (8.82)	‐	18 (28.57)
Hypomethylating agent	33 (4.67)	–	17 (27.87)	–	–	–	1 (0.53)	–	15 (19.23)	–
COVID‐19 treatment										
Oxygen support	365 (65.30)	2 (100)	17 (73.92)	27 (69.23)	2 (100)	2 (100)	5 (100)	157 (57.72)	–	–
HCQ	1571 (69.30)	2 (100)	5 (41.67)	3 (100)	15 (83.33)	2 (100)	2 (100)	173 (70.04)	–	–
Antibiotics	44 (46.81)	2 (100)	2 (22.22)	1 (100)	–	1 (100)	1 (100)	36 (45.57)	–	–
Corticosteroids	429 (42.52)	1 (100)	3 (75)	1 (100)	–	1 (100)	1 (100)	93 (41.33)	–	–
LPV/r	300 (22.66)	2 (50)	4 (33.33)	2 (100)	3 (16.67)	2 (100)	–	–	–	–
Clinical manifestations										
Fever	562 (74.24)	15 (71.42)	20 (90.91)	33 (82.5)	2 (100)	2 (100)	6 (84.71)	78 (75.73)	–	–
Cough	508 (67.64)	11 (50)	12 (5.89)	32 (82.05)	2 (100)	1 (100)	5 (83.34)	73 (70.87)	–	–
Dyspnea	155 (42.34)	1 (6.25)	4 (36.36)	19 (48.71)	–	–	4 (80)	33 (41.25)	–	–
Diarrhea	48 (15.53)	1 (6.25)	3 (60)	5 (15.15)	–	–	–	3 (33.33)	–	–
Respiratory distress	9 (17.65)	6 (37.5)	1 (33.33)	–	–	–	–	1 (100)	–	–
Laboratory findings ‐ *increase*										
LDH	16 (100)	1 (100)	2 (100)	3 (100)	2 (100)	2 (100)	4 (100)	–	–	–
CRP	242 (58.03)	1 (100)	4 (100)	5 (100)	1 (100)	2 (100)	5 (100)	9 (69.23)	–	–
D‐dimer	189 (49.22)	‐	3 (100)	4 (100)	2 (100)	1 (100)	2 (100)	3 (100)	–	–
Ferritin	134 (35.08)	1 (100)	4 (100)	4 (100)	1 (100)	1 (100)	1 (100)	2 (100)	–	–
Laboratory findings – *decrease*										
Hb	29 (90.62)	2 (100)	5 (62.5)	5 (100)	1 (100)	‐	5 (100)	10 (100)	–	–
PLT	31 (86.11)	3 (100)	7 (70)	2 (100)	1 (100)	1 (100)	3 (100)	–	–	–
WBC	50 (51.55)	2 (100)	3 (75)	4 (100)	–	–	4 (100)	23 (34.33)	–	–
Lymphocyte	218 (40.52)	1 (100)	2 (100)	3 (100)	–	2 (100)	6 (100)	14 (19.72)	–	–
Gender										
Male	962 (56.65)	20 (58.82)	21 (65.62)	24 (60)	8 (44.44)	13 (61.90)	61 (53.98)	158 (58.09)	51 (65.38)	43 (68.25)
Female	736 (43.35)	14 (41.18)	11 (34.38)	16	10 (55.56)	8 (38.01)	52 (46.02)	114 (41.91)	27 (34.62)	20 (31.75)

Abbreviations: ALL, acute lymphoblastic leukemia; AML, acute myeloid leukemia; CLL, Chronic lymphocytic leukemia; CML, chronic myelogenous leukemia; CRP, C‐reactive protein; HCQ, hydroxychloroquine; HL, Hodgkin lymphoma; LDH, lactate dehydrogenase; LPV/r, lopinavir/ritonavir; MM, multiple myeloma; PLT, platelet.

### 3.3 Treatments for COVID‐19 and hematological malignancies

The treatment options for COVID‐19 patients with hematological malignancies are summarized in Tables 2 and 3. Hydroxychloroquine (69.3%), oxygen therapy (65.3%), and anticoagulant therapies (64.0%) such as enoxaparin and heparin were the most administrated treatments options for COVID‐19. On the contrary, proteasome inhibitors (30.71%) and chemotherapy (23.46%) were the most used therapeutics to cure different types of blood cancer among patients with hematological malignancies.

### 3.4 Comorbidities and mortality rate

**FIGURE 2:**
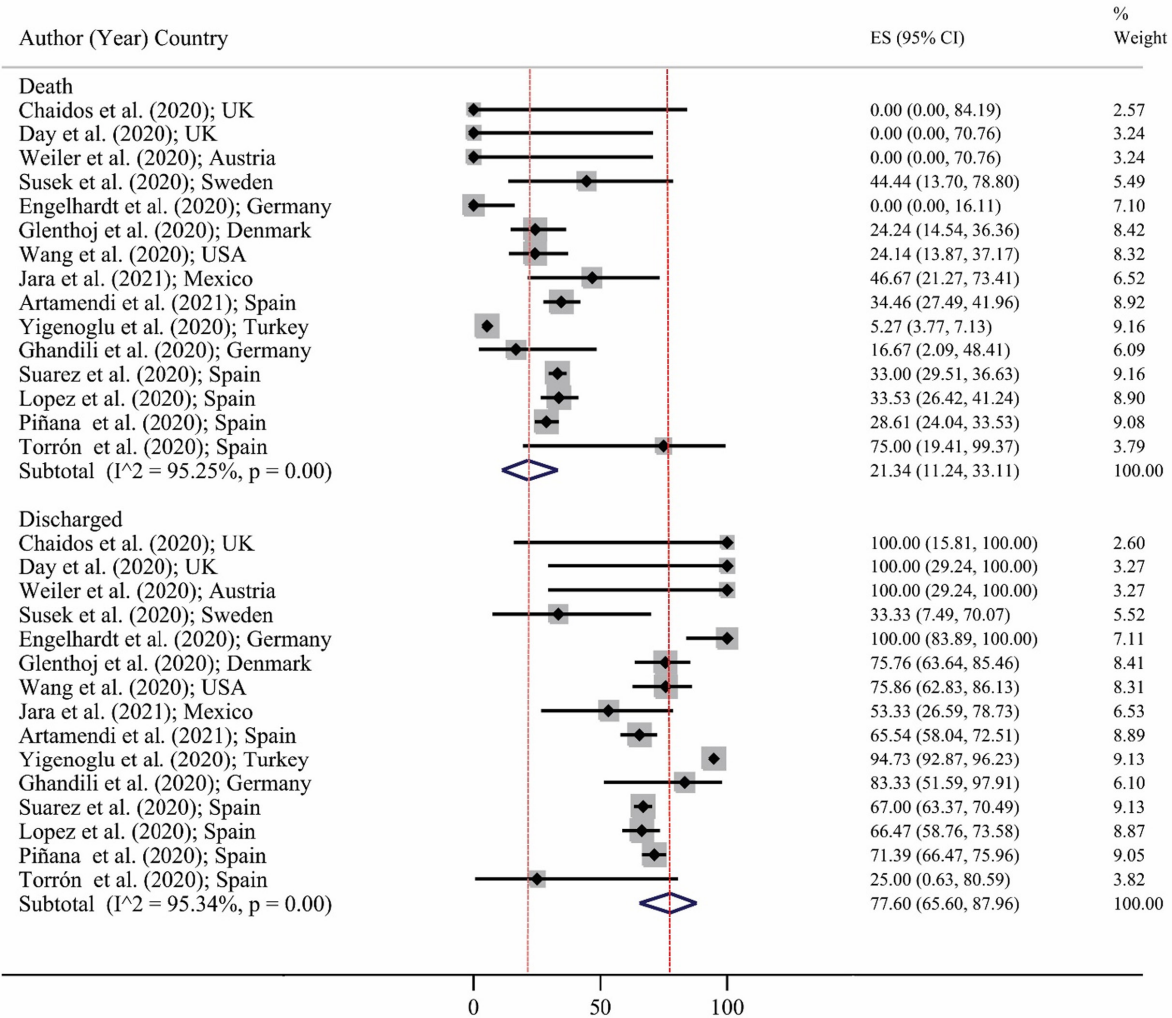
Forest plot for the death and discharge percent in patients with COVID‐19 and malignancy based on a random‐effects model. Each study identifies by the first author (year) and country. Each line segment's midpoint shows the percent estimate, length of line segment indicates 95% CI in each study, and diamond mark illustrates the pooled estimate in each subgroup

**FIGURE 3:**
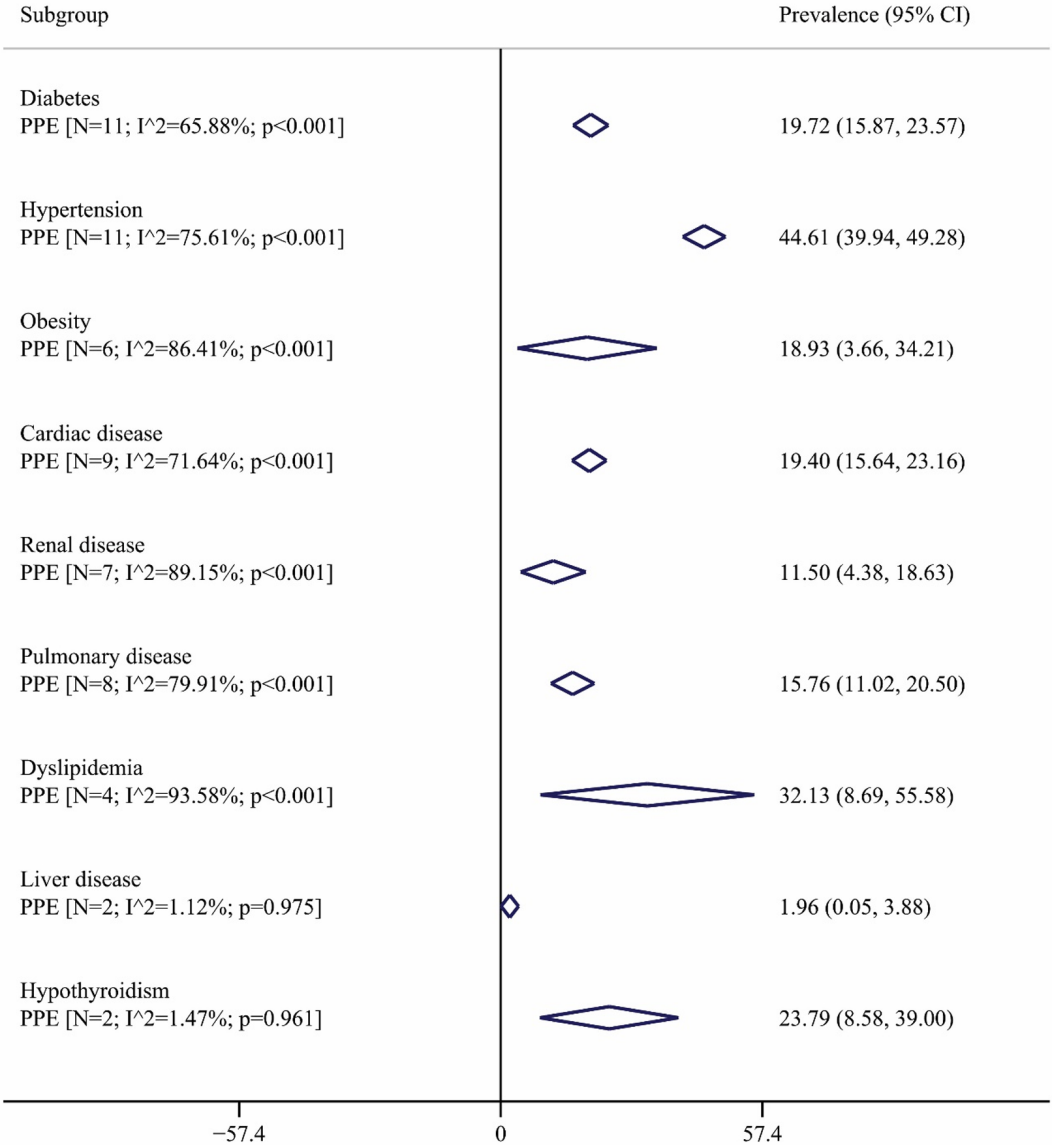
Pooled prevalence with 95% CI and heterogeneity indices of different comorbidity percent in patients with COVID‐19 and malignancy. The diamond mark illustrates the pooled percent, and the length of the diamond indicates the 95% CI. N is the number of the study in the analysis

**FIGURE 4:**
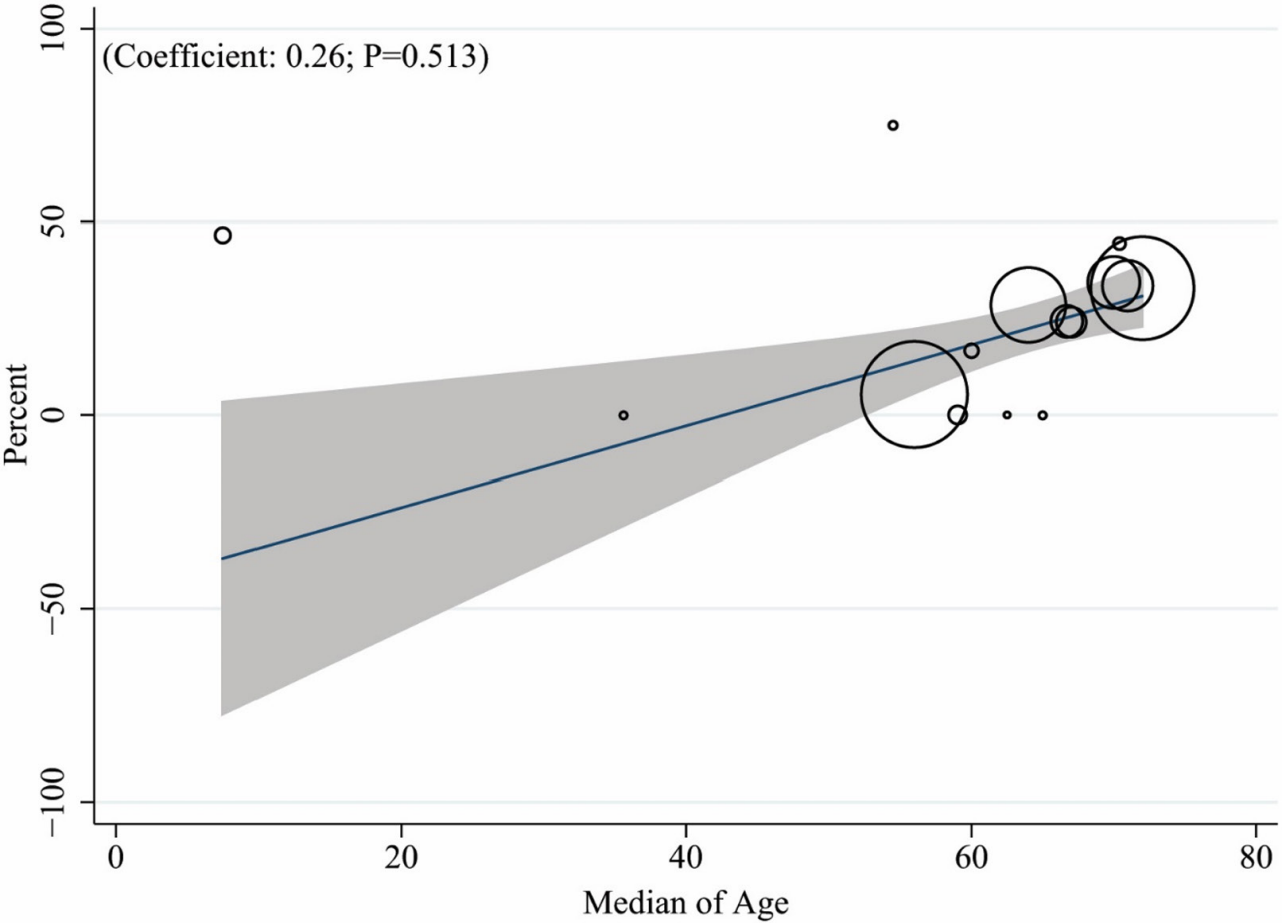
Association among death rate and Median of age by means of meta‐regression. The size of circles indicates the precision of each study. There is no significant association with respect to the death rate with Median of age

Figure 2 shows a forest plot for the mortality rate in COVID‐19 patients with hematological malignancies. Based on a random‐effects model, the pooled estimate of death and discharge percent were 21.34% (95% CI: 11.24 to 33.11) and 77.60% (95% CI: 65.60 to 87.96), respectively. It means that more than three‐quarters of cases with COVID‐19 and hematological malignancy were survived during hospitalization. In addition, Figure 3 shows the prevalence of comorbidities among patients with hematological malignancies and SARS‐CoV‐2 infection. The most prevalent comorbidity was hypertension (44.61%; 95% CI: 39.94 to 49.28), and the less one was liver disease (1.96%; 95% CI: 0.05 to 3.88). The prevalence of other comorbidities is shown also in Figure 2. We were interested in assessing the effect of age on the death rate in patients with COVID‐19 and malignancy (Figure 4). The meta‐regression demonstrated no significant association between death rate with age (*p* = 0.513).

### 3.5 Risk of bias assessment

The results of the critical appraisal (JBI checklist) of included studies are summarized in Table S1. Overall, 53 articles were identified as having a low risk of bias (quality assessment score > 7).

## 4 DISCUSSION

Since the onset of the COVID‐19 outbreak, several studies have reported the effects of COVID‐19 on cancer patients. In this regard, there is growing evidence that patients with a history of cancer have a higher rate of COVID‐19 mortality than individuals without cancer. In addition, it has been reported that patients with hematological cancers had the highest frequency of major adverse events.^5^, ^12^

Yeo et al. indicated that the cancer was associated with a 2.84‐fold increased risk of severe illness in COVID‐19 patients and a 2.60‐fold increased risk of death.^13^ The prevalence of cancer in COVID‐19 patients is very low. In a recent study, the pooled prevalence of cancer in COVID‐19 patients was 2%.^14^, ^15^

Also, the results of the same studies from China and the United States of America reported that about 1–2% and 6% of COVID‐19 patients had cancer, respectively.^14^, ^16^

The prevalence of hematological malignancies among COVID‐19 patients has not yet been well studied. In a related study conducted by Yigenoglu et al. in Turkey, it has been reported that 0.39% of the COVID‐19 patients had hematological malignancy. The most common hematological malignancies were non‐Hodgkin lymphoma (30.1%) followed by myelodysplastic syndromes (19.7%). They reported that about 5.27% of the patients have died.^17^

In the other study conducted by Mehta et al.^18^ in New York, the mortality rate in lymphoid neoplasms was higher than the myeloid malignancies (35% vs. 43%). Our results estimated that the mortality rate in COVID‐19 patients with hematological malignancies was 21.34%. This discrepancy observed in the results of these studies can be due to different sizes of studies. Therefore, the results of studies conducted in all areas highlight the urgent need to pay special attention to patients with hematologic malignancy infected with COVID‐19.

The progression of blood malignancies is usually accompanied by a weakening of the immune system, which is initiated by the disease and continues through the strategy of anti‐tumor therapies such as chemotherapy and radiation therapy Therefore, the suppressed immune system may lead to a greater vulnerability of cancer patients to COVID‐19.

A previous study showed that anti‐tumor therapy increased the risk of dangerous symptoms within 14 days of the diagnosis of COVID‐19 and recommended that cancer patients with COVID‐19 avoid treatments that suppress the immune system.^19^

On the hand, cytotoxic chemotherapies cause neutropenia and lymphocytopenia that aggravate the immunosuppressive status. This status leads to high infection rates and poor prognosis.^20^, ^21^, ^22^

There is currently no advice on the effectiveness of conventional and targeted treatment strategies in these patients.^23^

Hence, the risk–benefit ratio of these treatment strategies remains a challenge. In this regard, it has been demonstrated that radiation therapy has no higher risk of severe events related to the COVID‐19 for these patients.^5^

For example, Krengli et al.^24^ reported that radiation therapy could be considered a treatment strategy in COVID‐19 patients affected by myeloma. Recently, Liu et al.^25^ demonstrated that patients with hematological malignancies were at a higher risk of death if they received chemotherapy 3 months before the COVID‐19 diagnosis.

It is recommended that the cancer treatment strategies be postponed until the radiological and clinical symptoms of COVID‐19 have been completely disappeared.^26^, ^27^ These clinical symptoms were previously mostly treated by hydroxychloroquine. However, recently, WHO recommended healthcare systems cease the use of this drug.^28^, ^29^

The most common complications of COVID‐19 are fever, dyspnea, cough, muscle ache, confusion, headache, pneumonia, acute respiratory distress, and acute respiratory failure.^30^

The findings of the present study show that the highest incidence of clinical manifestations in patients with hematologic malignancy infected with SARS‐CoV‐2 belonged to fever (74.24%), cough (67.64%), fatigue (53.19%), dyspnea (42.47%), myalgia (20.50%), and the respiratory distress (17.65%). These results are consistent with those of other studies and confirm that pulmonary symptoms are the main clinical manifestations of COVID‐19 in more than half of the patients treated for the hematologic malignancies.^31^

Our results demonstrated that the most common comorbidities in patients with COVID‐19 and hematological malignancies were hypertension (44.61%) and dyslipidemia (32.13%). In addition, it revealed that the patients who died had more comorbidities. Also, it has been shown that the mortality rate of these patients is related to the disease status, the status of the immune system, and the level of inflammation.

The elevated levels of C‐reactive proteins were observed in 58.03% of patients with hematologic malignancy infected with SARS‐CoV‐2. Also, other laboratory findings such as the increased d‐dimer levels (49.22%), neutropenia (19.52%), and the increase in bilirubin levels were seen in these patients. It seems that laboratory findings on admission can help predict the severity of COVID‐19 in patients with hematologic malignancy. Furthermore, it has been demonstrated that the monitoring of RNA load in plasma can be useful to anticipate the COVID‐19 outcomes in these patients. Ghandili et al.^32^ reported that the increasing RNA titer is associated with the fatal outcomes in patients with acute myeloid leukemia infected SARS‐CoV‐2.

There are several limitations to this study. First, as our search was restricted to articles published in English, we might have missed some relevant publications in other languages. Second, only case series and research articles were enrolled in the meta‐analysis. Therefore, the existence of publication bias should be considered. Third, this study included patients whose RT‐PCR tests were positive for SARS‐CoV‐2. However, it is confirmed that false‐negative and false‐positive RT‐PCR may occur due to low amounts of SARS‐CoV‐2 concentrations and cross‐reaction with something that’s not SARS‐CoV‐2, respectively. Forth, heterogeneity in the study population selection and the retrospective characteristics was observed in the studies. Although the random‐effects model was assumed to reflect the similarity, there may still be differences of opinion. Fifth, all included studies have reported hospitalized patients. Due to these cases usually having a severe or moderate stage of disease, mild cases may be missed.

## 5 CONCLUSIONS

In this study, we reviewed the literatures reporting the COVID‐19 outcomes in patients with hematological malignancies. Our study reveals that about one‐quarter of patients with COVID‐19 and hematological malignancy have died during hospitalization. One of the most important reasons that confirm these patients are more vulnerable is their immune system dysfunction. Furthermore, anti‐cancer therapies may worsen their conditions. Therefore, the management of COVID‐19 in patients with hematological malignancies requires much more attention.

## CONFLICT OF INTEREST

The authors declare that they have no competing interests.

## AUTHOR CONTRIBUTIONS

Adel Naimi, Ilya Yashmi, Reza Jebeleh, Mohammad Imani Mofrad, Shakiba Azimian Abhar, Yasaman Jannesar, and Mohsen Heidary contributed in revising and final approval of the version to be published. All authors agreed and confirmed the study for publication.

## Supporting information

Table S1

## Data Availability

The authors confirm that the data supporting the results of this study is available within the article.
