# Supplementary material for: Comorbidities and mortality rate in COVID‐19 patients with hematological malignancies: A systematic review and meta‐analysis
Source: J Clin Lab Anal. 2022 Apr 6;36(5):e24387. doi: 10.1002/jcla.24387 (PMC9102765; doi:10.1002/jcla.24387)
Supplement: Supplementary file 1 — Table S1 [file JCLA-36-e24387-s001.docx]

***Supplement table 1. Quality assessment for validation studies on prevalence of comorbidities and mortality rate in COVID-19 patients with hematological malignancies***

| First author | Was the sample representative of the target population? | Were study participants recruited in an appropriate way? | Was the sample size adequate? | Were the study subjects and the setting described in detail? | Was the data analysis conducted with sufficient coverage of the identified sample? | Were objective, standard criteria used for the measurement of the condition? | Was the condition measured reliably? | Are all important confounding factors/subgroups/differences identified and accounted for? | Were subpopulations identified using objective criteria? | Was there appropriate statistical analysis? |
| --- | --- | --- | --- | --- | --- | --- | --- | --- | --- | --- |
| Santana | Yes | No | No | Yes | Yes | Yes | Yes | No | No | Yes |
| Ali | Yes | Yes | Yes | Yes | Yes | Yes | Yes | Yes | Yes | Yes |
| Nesr | Yes | No | Yes | Yes | Yes | Yes | Yes | Yes | Yes | Yes |
| Molina-Cerrillo | No | No | Yes | Yes | Yes | Yes | Yes | No | Yes | Yes |
| Largeaud | No | No | Yes | Yes | Yes | Yes | Yes | No | Yes | Yes |
| Bolaman | Yes | Yes | Yes | No | Yes | Yes | Yes | No | No | Yes |
| Pasin | Yes | Yes | Yes | No | Yes | Yes | Yes | No | No | Yes |
| Ibrahim | Yes | Yes | Yes | Yes | Yes | Yes | Yes | No | No | Yes |
| Chaidos | Yes | Yes | Yes | Yes | Yes | Yes | Yes | Yes | Yes | Yes |
| O’Kelly | Yes | No | No | Yes | Yes | Yes | Yes | No | No | Yes |
| Day | Yes | Yes | Yes | Yes | Yes | Yes | Yes | No | No | Yes |
| Bellmann-Weiler | Yes | No | Yes | No | Yes | Yes | Yes | No | No | Yes |
| Susek | Yes | No | Yes | No | Yes | Yes | Yes | No | No | Yes |
| Ye | Yes | No | No | Yes | Yes | Yes | Yes | No | No | Yes |
| Phillips | No | Yes | Yes | Yes | Yes | Yes | Yes | No | No | Yes |
| Zamani | Yes | Yes | Yes | No | Yes | Yes | Yes | No | No | Yes |
| Krengli | Yes | Yes | Yes | Yes | Yes | Yes | Yes | No | Yes | Yes |
| Kohla | Yes | Yes | Yes | Yes | Yes | Yes | Yes | Yes | Yes | Yes |
| Engelhardt | No | No | Yes | Yes | Yes | Yes | Yes | No | No | Yes |
| Rusconi | Yes | Yes | Yes | Yes | Yes | Yes | Yes | No | No | Yes |
| Denis | No | No | No | Yes | Yes | Yes | Yes | Yes | Yes | Yes |
| Moore | Yes | Yes | Yes | No | Yes | Yes | Yes | No | No | Yes |
| Vardanyan | Yes | Yes | Yes | Yes | Yes | Yes | Yes | No | No | Yes |
| Abdalhadi | Yes | No | No | Yes | Yes | Yes | Yes | Yes | Yes | Yes |
| Giammarco | Yes | No | Yes | No | Yes | Yes | Yes | No | No | Yes |
| Li | Yes | Yes | No | Yes | Yes | Yes | Yes | Yes | Yes | Yes |
| Marcia |  |  |  |  |  |  |  |  |  |  |
| Kamit | Yes | Yes | Yes | Yes | Yes | Yes | Yes | No | Yes | Yes |
| Otsuka | Yes | Yes | Yes | Yes | Yes | Yes | Yes | Yes | Yes | Yes |
| Bellesso | No | No | Yes | Yes | Yes | Yes | Yes | No | No | Yes |
| Glenthøj | Yes | Yes | Yes | Yes | Yes | Yes | Yes | No | No | Yes |
| Wang | Yes | No | Yes | Yes | Yes | Yes | Yes | Yes | Yes | Yes |
| Sánchez-Jara | No | No | Yes | Yes | Yes | Yes | Yes | No | Yes | Yes |
| Garcia‑Suarez | No | No | Yes | Yes | Yes | Yes | Yes | No | Yes | Yes |
| Martinez-Lopez | Yes | Yes | Yes | No | Yes | Yes | Yes | No | No | Yes |
| Regalado-Artamendi | Yes | No | Yes | Yes | Yes | Yes | Yes | Yes | Yes | Yes |
| Yigenoglu | No | No | Yes | Yes | Yes | Yes | Yes | No | Yes | Yes |
| Piñana | No | No | Yes | Yes | Yes | Yes | Yes | No | Yes | Yes |
| de la Cruz‐Benito | Yes | Yes | Yes | No | Yes | Yes | Yes | No | No | Yes |
| Başcı | Yes | Yes | Yes | No | Yes | Yes | Yes | No | No | Yes |
| Naseri | Yes | Yes | Yes | Yes | Yes | Yes | Yes | No | No | Yes |
| Song | Yes | No | Yes | Yes | Yes | Yes | Yes | Yes | Yes | Yes |
| Li | No | No | Yes | Yes | Yes | Yes | Yes | No | Yes | Yes |
| Baldacini | No | No | Yes | Yes | Yes | Yes | Yes | No | Yes | Yes |
| Farmer | Yes | Yes | Yes | No | Yes | Yes | Yes | No | No | Yes |
| Puyo | Yes | No | Yes | Yes | Yes | Yes | Yes | Yes | Yes | Yes |
